# Supplementary material for: Quantitative assessment of the relationship between behavioral and autonomic dynamics during propofol-induced unconsciousness
Source: PLoS One. 2021 Aug 11;16(8):e0254053. doi: 10.1371/journal.pone.0254053 (PMC8357089; doi:10.1371/journal.pone.0254053)
Supplement: S1 Appendix — (PDF) [file pone.0254053.s001.pdf]

S1 Appendix: Supplementary Information on Point Process Frameworks Used

Title: Quantitative assessment of the relationship between behavioral and autonomic dynamics during propofol-induced unconsciousness

Authors: Sandya Subramanian*, Patrick L. Purdon, Riccardo Barbieri, Emery N. Brown

*Corresponding Author

E-mail: sandya@mit.edu

Part A: Pulse Selection for EDA

The method we developed to select pulses from EDA data has been published previously [1-4]. The underlying principle is that the production and release of sweat from the sweat gland follows an integrate-and-fire process that can be modeled as a Gaussian random walk with linear drift. Therefore, the inter-pulse intervals should follow an inverse Gaussian distribution. Our previous work has showed that the inter-pulse intervals of EDA data follow inverse Gaussian and inverse-Gaussian like distributions that allow for slightly heavier or lighter tails to account for the aggregation of hundreds of sweat glands. This includes the lognormal distribution, which has a heavier tail than the inverse Gaussian [2,3]. This is likely due to the fact that observational EDA data is often characterized by periods of low activity, in which pulses are sparse and inter-pulse intervals are long, punctuated by bursts of high activity. Based on this result, we designed a systematic strategy to use the tail behavior of different distributions to select pulses from EDA data [1,4].

In this process, first we screen across a range of prominence thresholds, from 0.001 to 0.08, applied to phasic EDA data (after removing the slow-moving baseline or tonic component). The prominence of a peak quantifies how much taller it is than the lowest valleys on either side separating it from any peak of a commensurate height. At each prominence threshold, we can extract a set of pulses and compute the goodness-of-fit of several models to the inter-pulse interval distribution using the Kolmogorov-Smirnov distance (KS-distance). These models include the inverse Gaussian and lognormal, as well as distributions that typically have much lighter tails in the case of EDA data, such as the gamma and exponential. We use these KS-distances to select the optimal prominence threshold. The selected pulses should have an inter-pulse interval distribution that follows an inverse Gaussian or similarly heavier-tailed model like the lognormal, while not following lighter tailed models that are more likely to capture noise, such as the gamma and exponential. In other words, the KS-distances of the lognormal and

inverse Gaussian models should be under the significance cutoff, meaning that they are not significantly different from the data, while the KS-distances of the gamma and exponential should be above the cutoff, meaning that they are significantly different from the data. Using this process, we extracted pulses from phasic EDA data for all subjects in this study.

Part B: Point Process History-dependent Inverse Gaussian Models for HRV and EDA

After extracting R peaks from ECG and pulses from EDA, in both cases, we fit point process history-dependent inverse Gaussian models [5,6]. In both cases, since we hypothesize the intervals between events to follow an inverse Gaussian distribution, these point process models estimate the density function for the length of the next interval in continuous time. This density takes the form of an inverse Gaussian distribution; however, of the mean of the distribution has an autoregressive structure that depends on the previous p intervals. This autoregressive structure is the source of the history dependence and the order p is one of the hyperparameters of the model that is fit. From this density, the instantaneous mean and standard deviation of the intervals can be computed, and by simple univariate transformation of density, the instantaneous mean and standard deviation of the inverse, proportional to rate (heart rate or pulse rate). In the HRV model, the instantaneous estimates of autoregressive coefficients allow for dynamic estimation of the spectral content.

Since autonomic dynamics are not stationary over time, all of the parameters of the model, which include the p autoregressive coefficients and the shape parameter of the inverse Gaussian distribution, are re-estimated for every window of w seconds by maximizing the local likelihood within that window. The window length w is the second hyperparameter of the model that is fit. The values of p and w that

are screened differ between the HRV and EDA models. For the HRV model, based on previous work, the value of p is chosen from $\{6,8,10,12\}$ and the value of w from $\{60,90,120\}$. For the EDA model, where pulses are sparser in comparison, the value of p is chosen from $\{1,2,3\}$ and the value of w from $\{300,360,420,480,540,600,660,720,750\}$. Hyperparameter values are chosen based on screening across combinations of hyperparameters and choosing the one with the lowest KS-distance for the time-rescaled intervals [7].

References

1. Subramanian S, Barbieri R, Brown EN. A systematic method for preprocessing and analyzing electrodermal activity. Proc. 41st IEEE International Conf on Eng in Biol and Med (EMBC). 2019 Jul.
2. Subramanian S, Barbieri R, Brown EN. A point process characterization of electrodermal activity. Proc. 40th IEEE International Conf on Eng in Biol and Med (EMBC). 2018 Jul.
3. Subramanian S, Barbieri R, Brown EN. Point process temporal structure characterizes electrodermal activity. PNAS. 2020 Oct;117(42):26422-26428. DOI: 10.1073/pnas.2004403117.
4. Subramanian S, Purdon PL, Barbieri R, Brown EN. A model-based approach for pulse selection from electrodermal activity. bioRxiv. 2020. DOI: 10.1101/2020.05.17.098129.
5. Point Process Models of Human Heart Beat Interval Dynamics [Internet]. Available from: http://users.neurostat.mit.edu/barbieri/pphrv
6. Barbieri R, Matten EC, Alabi AA, Brown EN. A point-process model of human heartbeat intervals: new definitions of heart rate and heart rate variability. Am. J. Physiol. Heart Circ. Physiol. 2005 Jan;288(1):H424–435.
7. Brown EN, Barbieri R, Ventura V, Kass R, Frank L. The time-rescaling theorem and its application to neural spike train data analysis. Neural Computation. 2001;14:325–346.
