# Supplementary material for: Quantitative assessment of the relationship between behavioral and autonomic dynamics during propofol-induced unconsciousness
Source: PLoS One. 2021 Aug 11;16(8):e0254053. doi: 10.1371/journal.pone.0254053 (PMC8357089; doi:10.1371/journal.pone.0254053)
Supplement: S2 Appendix — (PDF) [file pone.0254053.s002.pdf]

S2 Appendix: Supplementary Information on EDA Analysis

Title: Quantitative assessment of the relationship between behavioral and autonomic dynamics during propofol-induced unconsciousness

Authors: Sandya Subramanian*, Patrick L. Purdon, Riccardo Barbieri, Emery N. Brown

*Corresponding Author

E-mail: sandya@mit.edu

Hyperparameters

For each subject, two hyperparameters were screened for each of HRV and EDA: the autoregressive model order and window length for local likelihood parameter fitting. Table S1 summarizes the optimal hyperparameter values used.

Table S1. Optimal hyperparameter values by subject in point process HRV and EDA models

Subject	HRV Model Order	HRV Window Length (sec)	EDA Model Order	EDA Window Length (sec)
1	6	120	1	660
2	12	90	2	720
3	6	60	1	660
4	8	60	1	600
5	6	90	2	540
6	6	90	1	750
7	8	120	1	540
8	10	120	1	750
9	6	90	1	480

Additional Figures

Figures S1-S6 below show the pulse rate and amplitude information for the six subjects not shown in the main text.

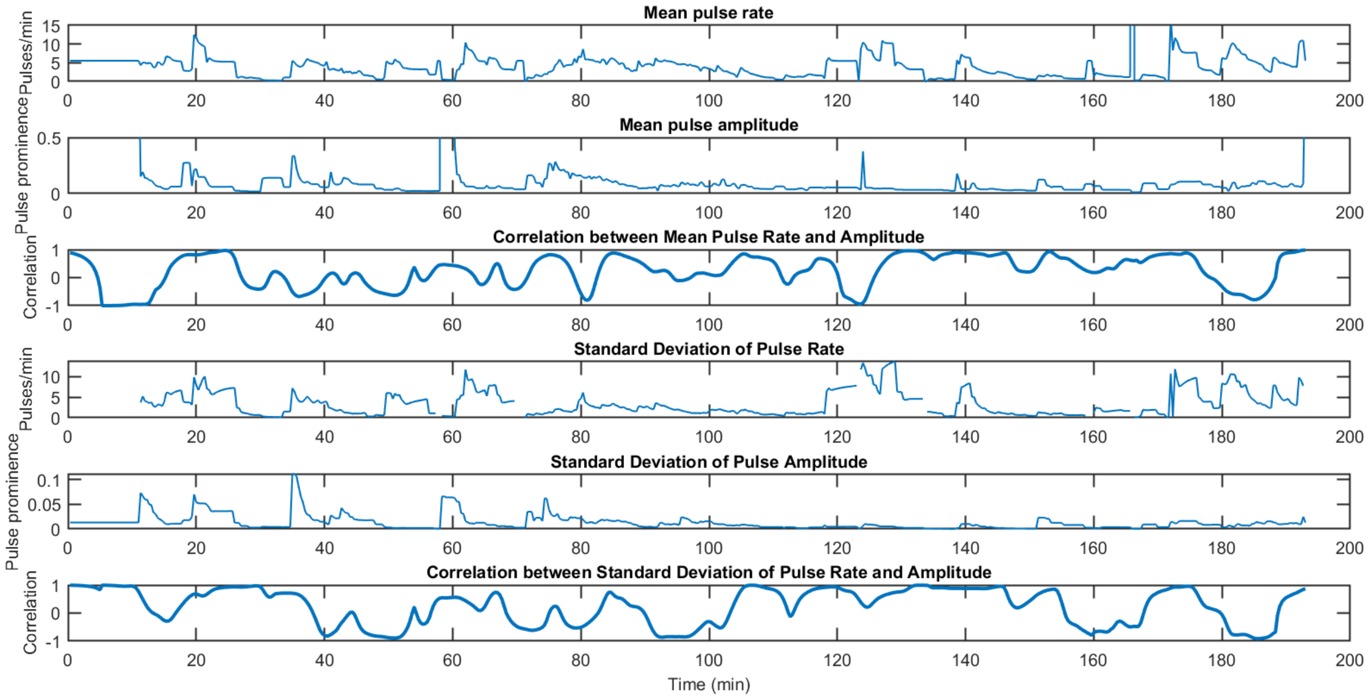

Fig S1. EDA indices from Subject 1. From top to bottom, mean pulse rate, mean pulse amplitude, correlation between mean pulse rate and pulse amplitude, standard deviation of pulse rate, standard deviation of pulse amplitude, and correlation between standard deviations of pulse rate and pulse amplitude.

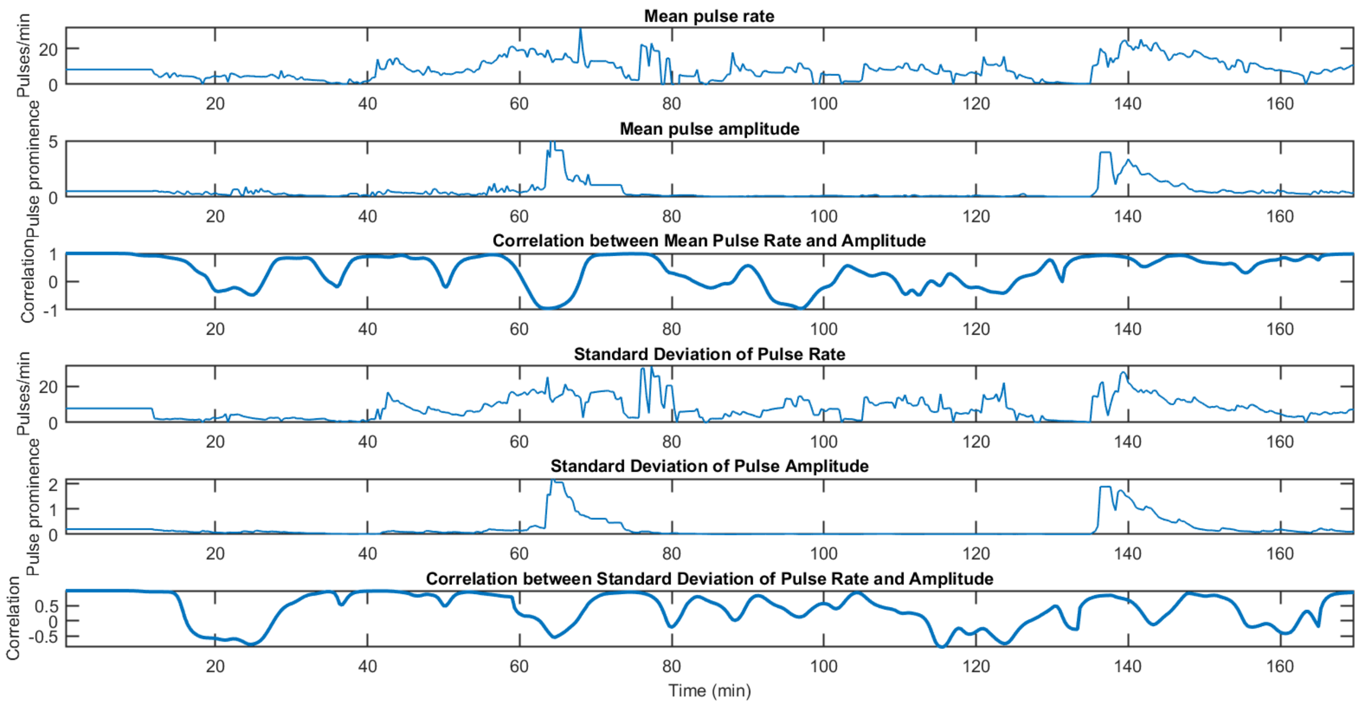

Fig S2. EDA indices from Subject 2.

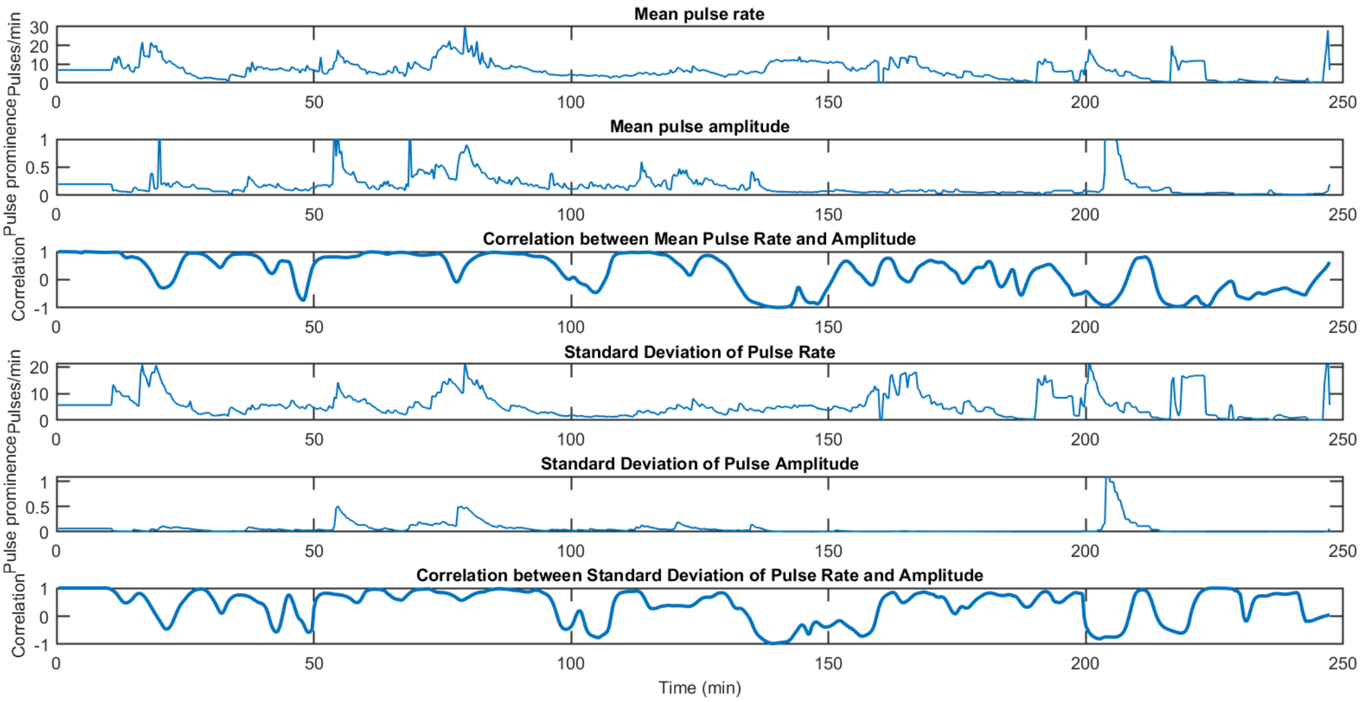

Fig S3. EDA indices from Subject 3.

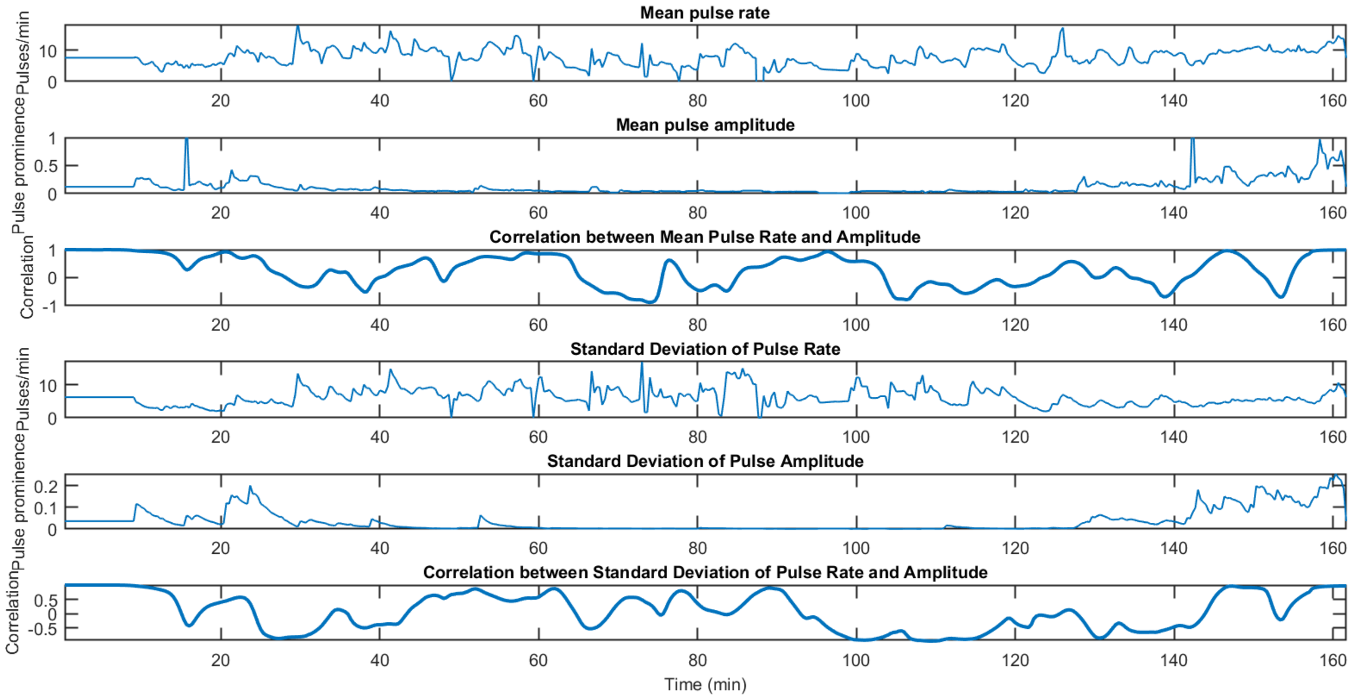

Fig S4. EDA indices from Subject 5.

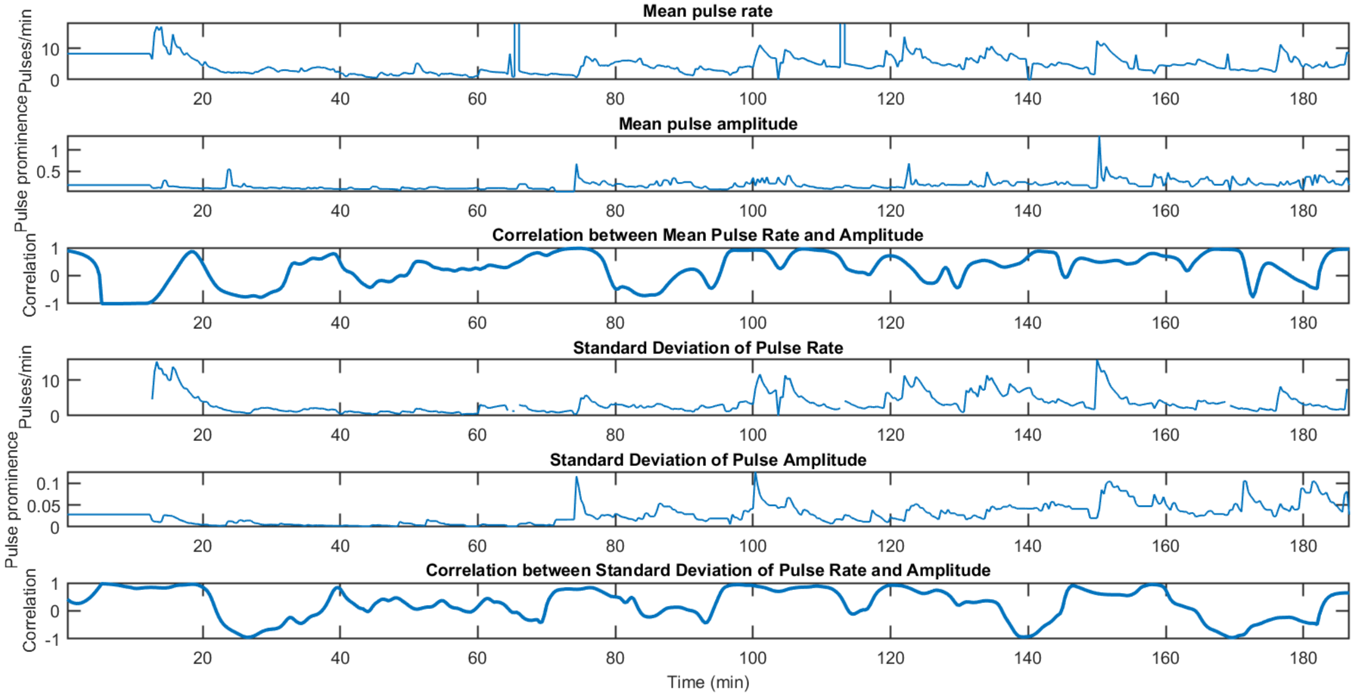

Fig S5. EDA indices from Subject 8.

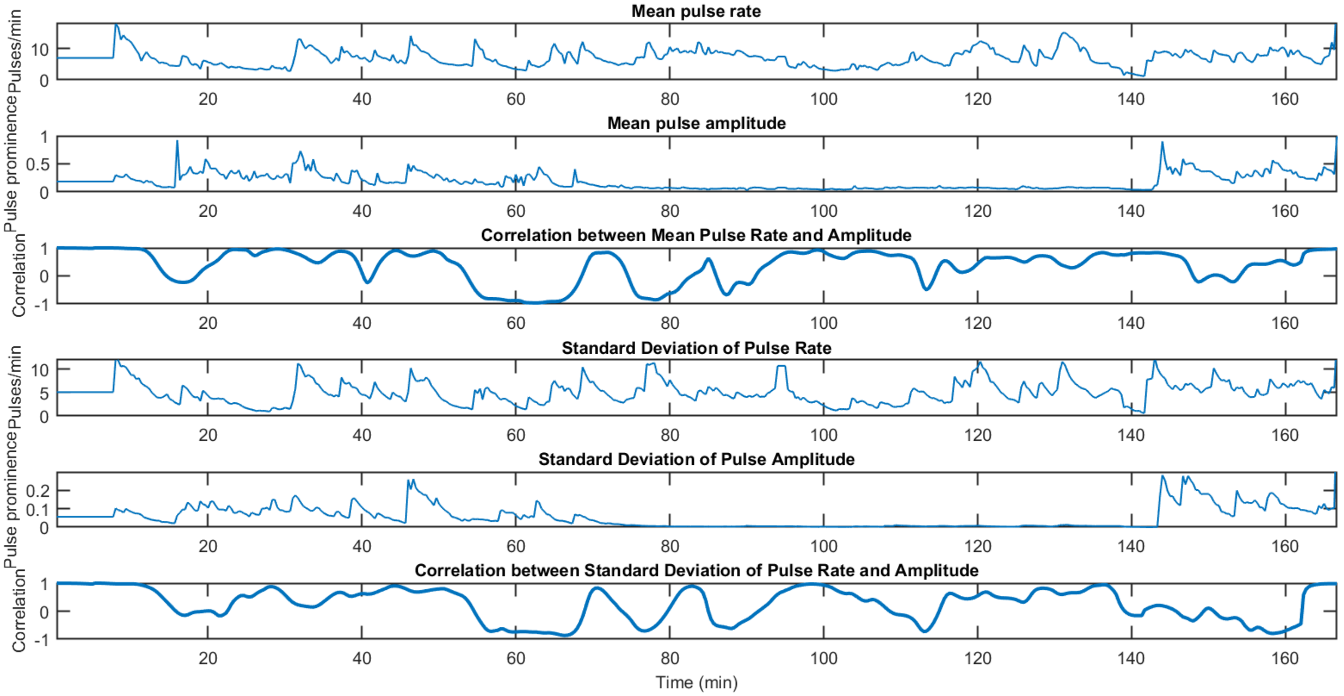

Fig S6. EDA indices from Subject 9.
