# Supplementary material for: Quantitative assessment of the relationship between behavioral and autonomic dynamics during propofol-induced unconsciousness
Source: PLoS One. 2021 Aug 11;16(8):e0254053. doi: 10.1371/journal.pone.0254053 (PMC8357089; doi:10.1371/journal.pone.0254053)
Supplement: S3 Appendix — (PDF) [file pone.0254053.s003.pdf]

S3 Appendix: Supplementary Information on Testing Logistic Regression Models for Questions 1-4

Title: Quantitative assessment of the relationship between behavioral and autonomic dynamics during propofol-induced unconsciousness

Authors: Sandya Subramanian*, Patrick L. Purdon, Riccardo Barbieri, Emery N. Brown

*Corresponding Author

E-mail: sandya@mit.edu

Part A: 95% Confidence Intervals for Differences in AUC values for Questions 1-4

Table S2 reports the 95% confidence intervals for the differences in AUC values between the multimodal and each of the unimodal models for Questions 1-4.

Table S2. 95% confidence intervals for differences in AUC values between multimodal and unimodal models.

	Multimodal vs HRV only model	Multimodal vs EDA only model
Question 1	(0.0245, 0.0287) *	(0.0864, 0.0953) *
Question 2	(-0.0410, -0.0322) *	(0.1169, 0.1363) *
Question 3	(0.0281, 0.0342) *	(0.1035, 0.1142) *
Question 4	(0.0527, 0.0741) *	(0.0385, 0.0629) *

*indicates statistical significance

Part B: Expanded results of regression models for Questions 1-4

Tables S3-S6 below show the results of testing a variety of combinations of hyperparameter values for each question.

Question 1

Table S3. Results of testing combinations of hyperparameter values for Question 1.

Metric within window	Window length (*w* sec)	History (*h* sec)	AUC (multimodal)
Mean	15	0	0.79
Mean	30	0	0.80
Mean	5	0	0.79
Mean	2	0	0.78
Mean	10	0	0.79
Median	30	0	0.80
Median	15	0	0.80
Median	15	60	0.83
Median	15	120	0.85
Median	10	120	0.84
**Median**	**20**	**120**	**0.86**
Mean	30	120	0.82
Median	30	120	0.85

Question 2

Table S4. Results of testing combinations of hyperparameter values for Question 2.

Metric within window	Window length (*w* sec)	History (*h* sec)	Time before and after (*t* min)	AUC (multimodal)
Median	15	N/A	15	0.74
Mean	15	N/A	15	0.74
Median	15	120	15	0.76
Mean	15	120	15	0.74
Median	10	N/A	15	0.72
Median	10	120	15	0.72
Median	5	N/A	15	0.72
Median	5	120	15	0.72
Median	2	N/A	15	0.71
Median	10	N/A	10	0.66
Median	30	N/A	15	0.74
Median	30	120	15	0.72
Median	20	120	15	0.71
Median	15	120	20	0.77
Median	30	120	20	0.77
Mean	30	120	20	0.76
Mean	15	120	20	0.76
**Median**	**20**	**120**	**20**	**0.78**
Mean	20	120	20	0.76

Question 3

Table S5. Results of testing combinations of hyperparameter values for Question 3.

Metric within window	Window length (*w* sec)	History (*h* sec)	Time before and after (*t* min)	AUC (multimodal)
Mean	15	N/A	15	0.53
Mean	30	N/A	15	0.51
Mean	5	N/A	20	0.54
Mean	30	N/A	20	0.53
Mean	30	N/A	10	0.54
Median	30	N/A	10	0.55
Median	5	N/A	20	0.57
Median	2	N/A	10	0.51
Median	10	N/A	5	0.47
Median	30	60	10	0.47
Median	30	60	20	0.61
Median	5	60	20	0.65
Median	5	120	20	0.72
Median	30	120	20	0.68
Median	20	120	20	0.72
Median	10	120	20	0.71
Median	15	120	20	0.71
Median	5	120	15	0.65
Median	20	120	15	0.65
Mean	20	120	20	0.72
Mean	5	120	20	0.69
**Median**	**20**	**240**	**20**	**0.83**
Median	30	240	20	0.78

Question 4

Table S6. Results of testing combinations of hyperparameter values for Question 4.

**Metric within window**	**Window length (*w* sec)**	**History (*h* sec)**	**AUC (multimodal)**
Median	30	120	0.40
Mean	30	120	0.60
Mean	15	60	0.66
Mean	10	60	0.64
Mean	5	N/A	0.70
Mean	10	N/A	0.72
**Mean**	**30**	**N/A**	**0.75**
Median	30	N/A	0.67
Mean	15	N/A	0.73
Mean	20	N/A	0.73
Mean	2	N/A	0.69
Mean	15	30	0.68
Mean	30	30	0.69

Part C: Calculation of Importance Scores for Each Index in the Final Models for Questions 1-4

Tables S7-S10 below shows the importance scores for each index for the best models for Questions 1-4. An individual feature was considered significant if it was included in the final models for at least 7 of the 9 subjects. We used 7 out of 9 subjects as the threshold to allow for variability in each of the leave-one-subject-out trained models since it is not guaranteed that the features in each model are identical. However, we still require a feature to be included in the final models of at least two-thirds of the subjects overall. Since several models included lagged time windows as history for each index, we report the number of features associated with each index (including history) which were included in the final models of at least 7 of the 9 subjects. We calculated the importance scores of each index based on the fraction of associated features that were included in the final model of at least 7 out of 9 subjects. The number of features associated with each index depends on the duration of history *h* included compared to the window length *w*. For example, if *w* is 20 seconds and *h* is 120 seconds, the total number of features associated with each index is 7, one for the current window and six time-lagged windows. If *h* is 240 seconds for the same window length *w*, there are six further time-lagged windows as features for each index. If no history is included in the model, each index results in one feature.

Question 1

Table S7. Importance scores for features in final models for Question 1.

Index	Modality	Multimodal models	HRV-only models	EDA-only models
muRR	HRV	6/7	6/7	

sigmaRR	HRV	6/7	6/7	
muHR	HRV	7/7	7/7	
sigmaHR	HRV	4/7	6/7	
Total power	HRV	2/7	3/7	
LF	HRV	3/7	6/7	
HF	HRV	3/7	3/7	
LF/HF	HRV	7/7	7/7	
LFnu	HRV	6/7	6/7	
HFnu	HRV	5/7	2/7	
Tonic_EDA	EDA	4/7		2/7
muPR	EDA	7/7		7/7
sigmaPR	EDA	7/7		7/7
mu_amp	EDA	6/7		4/7
sigma_amp	EDA	2/7		3/7

Question 2

Table S8. Importance scores for features in final models for Question 2.

**Index**	**Modality**	**Multimodal models**	**HRV-only models**	**EDA-only models**
muRR	HRV	5/7	5/7	
sigmaRR	HRV	0/7	4/7	
muHR	HRV	4/7	6/7	
sigmaHR	HRV	1/7	3/7	
Total power	HRV	2/7	2/7	
LF	HRV	1/7	4/7	
HF	HRV	2/7	3/7	
LF/HF	HRV	5/7	6/7	

LFnu	HRV	1/7	2/7	
HFnu	HRV	5/7	6/7	
Tonic_EDA	EDA	5/7		2/7
muPR	EDA	4/7		2/7
sigmaPR	EDA	1/7		7/7
mu_amp	EDA	5/7		7/7
sigma_amp	EDA	3/7		6/7

Question 3

Table S9. Importance scores for features in final models for Question 3.

**Index**	**Modality**	**Multimodal models**	**HRV-only models**	**EDA-only models**
muRR	HRV	5/13	7/13	
sigmaRR	HRV	3/13	3/13	
muHR	HRV	9/13	9/13	
sigmaHR	HRV	6/13	1/13	
Total power	HRV	10/13	12/13	
LF	HRV	11/13	12/13	
HF	HRV	9/13	10/13	
LF/HF	HRV	13/13	13/13	
LFnu	HRV	13/13	12/13	
HFnu	HRV	8/13	7/13	
Tonic_EDA	EDA	5/13		1/13
muPR	EDA	9/13		13/13
sigmaPR	EDA	10/13		9/13
mu_amp	EDA	12/13		3/13
sigma_amp	EDA	9/13		3/13

Question 4

Table S10. Importance scores for features in final models for Question 4.

Index	Modality	Multimodal models	HRV-only models	EDA-only models
muRR	HRV	1	1	
sigmaRR	HRV	1	1	
muHR	HRV	1	1	
sigmaHR	HRV	1	1	
Total power	HRV	1	1	
LF	HRV	1	1	
HF	HRV	1	1	
LF/HF	HRV	1	1	
LFnu	HRV	1	1	
HFnu	HRV	1	1	
Tonic_EDA	EDA	1		1
muPR	EDA	1		1
sigmaPR	EDA	1		1
mu_amp	EDA	1		0
sigma_amp	EDA	1		0

Part D: Comparison of Point Process Features to Standard HRV/EDA features

We repeated the analysis for Question 1 using an alternative set of features which included standard non-point process HRV measures and non-point process EDA measures. These are detailed in Table S11 below. The standard HRV features were adopted from [1].

Table S11. Summary of standard autonomic indices for comparison

Index	***Modality***	***Description***
numBeats	HRV	Number of R complexes
RMSSD	HRV	Root mean square of differences between adjacent RR intervals
LF	HRV	Low freq. power, absolute (0.04-0.15 Hz), by performing spectral analysis of the tachogram
HF	HRV	High freq. power, absolute (0.15 – 0.4 Hz), by performing spectral analysis of the tachogram
LF	HRV	Low freq. power, absolute (0.04-0.15 Hz)
LF/HF	HRV	Ratio of LF/HF
LFnu	HRV	Low freq. power, normalized
HFnu	HRV	High freq. power, normalized

Index	***Modality***	***Description***
numPeaks	EDA	Number of EDA peaks, computed by setting a prominence threshold of 0.001 across all EDA data
meanHt	EDA	Mean prominence of EDA peaks
sigmaHt	EDA	Standard deviation of EDA peaks

We tested hyperparameter values for Question 1 using these features and compared them to the performance achieved using the same hyperparameter values and the point process features (taken from Table S3). To focus the comparison on the features alone, no models included any history. All models used median as the metric to consolidate information within each window. Table S12 shows the results of comparing standard features to the point process features. The StAR framework was used to compute 95% confidence intervals for the difference in AUC values between point process features and standard features [2]. A confidence interval that does not contain zero is statistically significant. The point process features outperform the standard features for every set of hyperparameters tested. Not only that, since the point process features are constructed to be instantaneous while the standard features are meant for longer periods of time (5 minutes to 24 hours), the performance of the standard features is not robust to short time windows.

Table S12. Results of testing combinations of hyperparameter values for Question 1 using both standard features and point process features

**Window length (*w* sec)**	**Best AUC using standard features**	**Best AUC using point process features**	**95% Confidence Interval for difference in AUC**
30	0.67	0.80	(0.1247, 0.1354) *

20	0.69	0.81	(0.1108, 0.1186) *
15	0.69	0.80	(0.1084, 0.1148) *
10	0.67	0.79	(0.1199, 0.1255) *
5	0.65	0.79	(0.1397, 0.1443) *
2	0.62	0.78	(0.1575, 0.1608) *

*indicates statistical significance

References

1. Malik M. Heart rate variability. Annals of Noninvasive Electrocardiology. 1996; 1(2): 151-181.
2. DeLong E.R., DeLong D.M., and Clarke-Pearson, D.L. Comparing the Areas Under Two or More Correlated Receiver Operating Characteristic Curves: A Nonparametric Approach. Biometrics, 1988; 44: 837-845.
